# New explainability method for BERT-based model in fake news detection

**DOI:** 10.1038/s41598-021-03100-6

**Published:** 2021-12-08

**Authors:** Mateusz Szczepański, Marek Pawlicki, Rafał Kozik, Michał Choraś

**Affiliations:** 1 ITTI Sp. z o.o., Poznań, Poland; 2 Bydgoszcz University of Science and Technology (PBS), Bydgoszcz, Poland

**Keywords:** Engineering, Mathematics and computing

## Abstract

The ubiquity of social media and their deep integration in the contemporary society has granted new ways to interact, exchange information, form groups, or earn money—all on a scale never seen before. Those possibilities paired with the widespread popularity contribute to the level of impact that social media display. Unfortunately, the benefits brought by them come at a cost. Social Media can be employed by various entities to spread disinformation—so called ‘Fake News’, either to make a profit or influence the behaviour of the society. To reduce the impact and spread of Fake News, a diverse array of countermeasures were devised. These include linguistic-based approaches, which often utilise Natural Language Processing (NLP) and Deep Learning (DL). However, as the latest advancements in the Artificial Intelligence (AI) domain show, the model’s high performance is no longer enough. The explainability of the system’s decision is equally crucial in real-life scenarios. Therefore, the objective of this paper is to present a novel explainability approach in BERT-based fake news detectors. This approach does not require extensive changes to the system and can be attached as an extension for operating detectors. For this purposes, two Explainable Artificial Intelligence (xAI) techniques, Local Interpretable Model-Agnostic Explanations (LIME) and Anchors, will be used and evaluated on fake news data, i.e., short pieces of text forming tweets or headlines. This focus of this paper is on the explainability approach for fake news detectors, as the detectors themselves were part of previous works of the authors.

## Introduction and rationale

### Definition of ‘fake news’

The term ‘Fake News’was popularised and politicised during the 2016 U.S. election^1^. It has become a ’buzzword’, used in contexts deviating from its previous definition^1,2^. Initially, it meant pieces of inaccurate news, often fabricated on purpose, mimicking in its form the news media content^1,3^. The term could also be used in the context of more specific, misleading content categories, such as satire, news parody, fabrication, manipulation, advertising, and propaganda^2^. The expression was also appropriated by politicians during the mentioned presidential campaign^1^ where it was applied to discredit legitimate news sources and information they convey, giving it an additional meaning^1,2^. In this paper, the term will be used to denote purposefully fabricated pieces of information that are presented as legitimate, setting the focus on the disinformative aspect^1,2^.

Fake news is generally created pecuniary or ideologically^4^. The first purpose utilises the fact that a traffic spike gained by the viral spread of fake news can generate considerable revenue from advertisements^4^. The second reason is more complex and depends on the agency of the author. For example, fake news can be created and spread to discredit one political option or aggrandise others^4^. Although its ultimate role and eventual effect on votes may be debatable^5^, fake news can have a negative impact on society and pose a real danger that should not be underestimated. For instance, it can contribute to the rising distrust in children vaccination^6^ or even lead to international tensions^7^. This fact has raised concerns of the world leaders^2^ and scientists, who now seek ways to find effective countermeasures against it^6^.

### Role of social media in fake news dissemination

Social media play an essential role in the spreading of fake news. Studies from the USA 2016 elections^4^ show that, on average, 41.8% of all traffic to fake news outlets during the period of 2016 U.S. election was generated through social media. In comparison, for genuine news sites average traffic share from this type of activity was equal only to 10.1%^4^. It is worth noting that this statistic does not show how many fake news headlines or ’tweets’ were just seen without clicking on the link. It is estimated that during the election period, every American adult encountered on average from 1 to 3 fake news articles^3,4^.

Several factors make social media convenient platforms for the spreading of fake news. One of the biggest ones is their size and reach. Facebook alone at the end of 2020 had around 2.8 billion users worldwide^8^. In the United States, 36% users claim that they get news from Facebook^9^. Generally, over 50% of adult Americans get their news from social media platforms at least every now and again^9^. It is a notable increase in comparison with the year 2017, for which this percentage was equal to 44%^10^. The tendency is currently increasing, and the role of social media as a news provider will probably become even more prominent in the upcoming future.

The other factors are the direct consequence of the nature of social media. They are a group of Internet-based services built on top of the ideological and technological foundations of allowing users to create and exchange content^11,12^. This core trait, in the context of the media and news distribution, has led to the rise of civilian journalism, where everyone can create or share journalistic outputs and reach mass audience^2^. Alas, they can do it without the fact-checking or third-party filtering present in typical media outlets^4^. Inversely, social media have become a platform for professional journalists as well who use them to engage with the audience and break down big news^2^. Inadvertently, this has blurred the idea of news sources^2^.

There is also the matter of how the algorithms present on those platforms work and what kind of behaviour they encourage. As highlighted by Ciampaglia^6^, they may reinforce cognition biases. For instance, bandwagon heuristic, which is the phenomenon where people tend to join what they perceive to be existing or expected majorities or dominant positions in society^13^, seems to be especially prevalent there^4^. Posts that are liked, shared, and commented are more likely to receive the user’s attention and therefore be spread further, which may propagate unverified information^2^. People on social media also tend to have ideologically segregated friend groups that match the given user worldview and are much more likely to believe and share content fitting their beliefs^4,14^. All of this may lead to the ’echo chamber’ effect. It is defined as ’creation of the environment in which the opinion, political leaning, or belief of users about a topic gets reinforced due to repeated interactions with peers or sources having similar tendencies and attitudes where similar convictions are being reinforced’^15^, p. 1. In such conditions, fake news is harder to verify and may lead to further polarization, and radicalization^3,4^.

Finally, the issue of prevalent social bots infesting those platforms should be brought up^3^. They can be used to rapidly disseminate fake news^3,12^ through manipulating algorithms present on social media and therefore utilising the sociological and psychological phenomena mentioned above. They are not fully autonomous and automatic yet; however, some researchers consider an eventuality when they reach such a level. Theoretically, the bots could, through the coordinated action of disinformation, cause social crises^12^. Defence mechanisms that can effectively filter content on a large scale are therefore needed.

### Contribution

The danger of fake news is clear, as is the need for explainable solutions that can reduce its presence in social media. Thus, the goal of the paper is to contribute to this effort by exploring a surrogate-type approach to explainability, in conjunction with the supervised BERT-based model tuned for fake news detection in a social media-like environment. The major contribution of this work is:

- The proposition of an approach providing explainability to the existing BERT-based fake news detectors without the need of extensive changes in deployed architecture,
- The verification of the usability of the two selected surrogate-type methods in the context of fake news detection, including Anchors, which, to the best of our knowledge, was used for the first time with this kind of fake news detectors,
- The execution of an experimental evaluation of the chosen methods in the introduced domain on two distinct architectures and datasets,
- Highlighting the potential improvements that can be made for this approach.

Therefore, the major contribution of this paper is not the fake news detection method itself, but the new innovative explainability solution for this task.

## Related works

### Fake news detection approaches

Potential threats of fake news have raised concerns^1,3,6^ and lead to the development of various countermeasures, some proposed and integrated by social media platforms themselves^3^.

 Broadly speaking, fake news detection tools and methods may be divided into one of the two main categories: network-based or linguistic-based^16,17^. However, hybrid approaches using elements from both groups are also present^16,17^.

Network-based approaches can estimate the truthfulness of news by assessing the veracity of the source. They utilise network properties such as, for example, authors, timestamps or included links^16,17^ and focus on either heterogeneous or homogeneous networks^18^. A heterogeneous network consists of various types of nodes, while the homogeneous ones are made of only one type^18^. An instance of this approach that investigates homogeneous networks is presented by Zhou, X. & Zafarani, R.^18^ who propose to represent news articles as a set of sociologically based patterns across different network levels. For instance, ’Farther-Distance Pattern’ represents the fact that ’Fake news spreads farther than true news’^18^, p. 51. To reflect such a pattern as a machine learning feature, geodesic and effective distances between nodes are calculated. Pure network-based approaches are relatively rare, and those techniques tend to be used as complementary for linguistic-based approaches^16,17^.

Contrary to the network-based approaches, linguistic-based methods focus on the content of the investigated news^16,17^. They are trying to find anomalies in the text to verify its legitimacy, under the idea that there exist certain patterns specific for fake news^16,17^. To illustrate, the unusually high frequency of some words may be a cue suggesting the abnormality of an investigated text. Methods that concentrate on assessing credibility through frequencies belong to the statistical analysis subcategory, and example of it can be found in the work of Ksieniewicz, P., Choraś, M., Kozik, R. & Woźniak, M.^19^. The solution presented there employs a count vectorizer to obtain occurrences of each word in the text and then uses an ensemble of decision trees to perform classification.

However, there are more subcategories within linguistic-based methods. There is sentiment analysis, in which the main goal is to verify if a text contains objective information, and if it does not, to decide how it is expressed^16,17,20^. This approach can be effectively used to detect fake news. Dickerson et al.^21^ used what they called tweet sentiment which in pair with their sentiment-aware architecture called SentiBot, was able to distinguish humans from bots.

The deep syntax analysis method is based on the Probability Context Free Grammars (PCFG). Through PCFG, the sentences are transformed into a set of rules representing their syntax structure, which are then further transformed into the parse tree with an assigned probability^17,22^. This obtained structure can then be compared with the patterns characterising false information and thus employed to fake news detection^17^. However, as Zhang et al.^16^ suggest, those are often designed to work with either unique data types or within predefined contexts, reducing their flexibility and usability on a broader scale. In the work of Iyengar et al.^23^, an instance of such an approach applied to the task of SPAM emails detection is presented.

A thorough mapping study of fake news detection approaches, including text analysis, NLP-based approaches, psycholinguistic features, syntax-based methods, non-linguistic methods, reputation analysis, network data analysis and more has been performed by Choraś, M. et al.^24^.

Furthermore, there are approaches which may not have been originally designed to detect fake news, but could be successfully applied to this task.

 Xu et al.^25^ proposed a solution to the problems occurring during an analysis of short texts, such as high degree of noise, usage of abbreviations, and introduction of new words. The microblog emotion classification model, CNN_Text_Word2vec, trains distributed word embeddings on each word and uses them as inputs for the model. These allow the model to learn microblog text features through parallel convolution layers with varying convolution kernels. This method reported a significantly higher accuracy than the competing methods in the evaluated task. Thus, it could also prove valuable for fake news detection in social media, especially for twitter-like content.

Another approach was presented by Tian et al.^26^, where the authors discuss the matter of abnormal behaviors detection of insiders to prevent urban big data leakage. The detection was achieved based on the characteristics of user’s daily activities, obtained from a combination of several deep learning models and three perspectives: feature deviation, sequence deviation and role deviation. The experimental results have proven the solution’s ability to capture behavioral patterns and detect any abnormalities. Even though the method was designed for another environment, it may be able to spot deviations in social media users’ actions. For instance, this could help uncover bots or stolen accounts.

Qiu et al.^27^ proposed a concept extraction method, called Semantic Graph-Based Concept Extraction (SGCCE), designed to extract the concepts in the domain of big data in smart cities. It uses the graph structure-based approach to utilize semantic information in an effective manner. This method can also find applications in fake news detection, especially with the network-based approaches.

### BERT-bidirectional encoder representations from transformers

One of the most prolific recent advances in natural language processing is Bidirectional Encoder Representations from Transformers (BERT)^28^. Since its proposition by Google researchers in October of 2018, it has had a notable impact on the field of NLP, outclassing other approaches used at that time^29^. Its success is the result of the Masked Language Model (MLM), which randomly masks tokens in the input and forces the model to predict the original ID based on the surroundings. It enables to jointly condition on both the left and right contexts and, consequently, to obtain a better word representation.

BERT-based models had already been successfully applied to the fake news detection task. For example, the work presented by Jwa et al.^30^ had used it to a significant effect. The proposed model, exBAKE, applied BERT for the first time in fake news detection using a headline-body dataset. BERT was pre-trained with additional data explicitly related to the news to better express the representation, and further fine-tuned with Linear and Softmax layers for classification. This approach achieved a better F1-score (F1) than the other state-of-the-art methods.

On the other hand, Kula et al.^29^ discussed a hybrid architecture mixing BERT with a Recurrent Neural Network (RNN). BERT performs the role of a word embedding layer, while the RNN on top of it is used for document embedding. Few variants were tested, and all achieved results were comparable with those for similar datasets. This work was further expanded by Kula et al.^31^ with the tests on the new variants of hybrid architectures.

Lastly, Kaliyar et al.^32^ present a system that utilises a combination of three parallel blocks of single-layer Convolutional Neural Networks (CNNs) together with BERT to achieve a score of 98.90% on the test data. BERT serves there as an embedding layer responsible for generating word representations. Its output is then processed by the mentioned blocks of CNNs, with each using different kernel sizes and filters supplemented with a max-pooling layer across each of them. As the authors highlight, it allowed for better semantic representation of words with varying lengths.

### The need for explainability

However, proposing a solution that is just efficient in detecting potential fake news is no longer enough^33^. Due to the increasing role and responsibility of artificial intelligence in modern society, concerns regarding its trustworthiness have been expressed^34,35^. Those come from the fact that most of the AI solutions employed, especially Deep Neural Networks (DNNs), are ’black-box’ type models^36,37^. It means that their complexity issuing from huge parameters space and combination of algorithms makes them uninterpretable for humans, i.e., the decision process cannot be fully comprehended^36^. Such a model can be full of biases, basing its decisions on unjust, outdated, or wrong assumptions, which can be overlooked with the classical approaches to the model effectiveness estimation^35^. Ultimately, this leads to the lack of trust in the opaque model^35,36^.

Therefore, to alleviate the issues present in the ’classical’ AI, explainable artificial intelligence methods are proposed^35^. As the Barredo Arrieta, A., et al.^36^ highlight, xAI proposes the development of machine learning techniques producing highly effective, explainable models which humans can understand, manage and trust. Therefore, it can be defined as the models ’which given an audience, produce details or reasons to make its functioning clear or easy to understand’^36^, p. 6. Thus, xAI can help to make the model more secure, less prone to errors, and more trustworthy.

Let us imagine a real user of a fake news detection system, especially in critical applications, like law enforcement or journalism. If certain fake news content was related to an activity classified as crime, the law enforcement (police) and forensics officer cannot just justify the initiated procedures by saying that the AI model/system told them to do so, they should be able to understand and easily interpret the outputs of the system. Therefore, the following work is motivated by this real demand of explainability and practical use of AI in fake news detection.

The need for explainability is also present in the natural language processing and the fake news detection domain. There already exist attempts to make processes occurring within BERT-based architectures transparent. For instance, exBERT^38^ is an interactive tool designed to help its user formulate a hypothesis about the model’s reasoning process. The offered interactive dashboard can provide an overview of both the model’s attention and internal representation.

An alternative tool, called visBERT, was proposed by van Aken et al.^39^. Based on the research suggesting that reliance on the attention mechanism may not be ideal for explanation purposes^40^, the authors devised an entirely different approach. Instead of using attention weights, visBERT follows the transformations performed on the tokens when they are processed by the network’s layers. The hidden states of each Transformer encoder blocks are extracted, and then, through the application of Principal Component Analysis (PCA)^41^ mapped to 2D space. There, the distance between tokens can be interpreted as semantic relations.

Furthermore, there is a noticeable interest in developing the explainable fake news detectors, both linguistic- and network-based. One of such initiatives is dEFEND^42^. It utilises co-attention component modelling relations between encoded news sentences and user comments to discover the top-k most explainable and check-worthy amongst them.

On the other hand, Propagation2Vec^43^ is a proposition of the propagation network-based technique for the early fake news detection methods. It effectively exploits patterns of news records propagation present, for example, in social media (which can be represented as trees composed of nodes and cascades) to assess their veracity. Moreover, the logic of the underlying hierarchical attention model can be explained with the analysis of the node-level and cascade-level attention weights.

The final example, xFake^44^, is an advanced architecture composed of three frameworks, each of them analysing a piece of news from different facets. At the same time, each framework is self-explainable through the application of different techniques. For instance, the PERT framework analyses news from the linguistic perspective, extracting linguistic features and training the XGBoost^45^ classifier on them. Then it uses a perturbation-based method to measure feature importance. This way, the solution provides explanations over many perspectives, extending them with supporting examples and visualisation.

## Proposed approach

### Method overview

The proposed approach to explainability of the BERT-based fake news detector is an alternative to the solutions listed in the previous section. It has, in comparison to the described methods, one crucial advantage. It can be rapidly deployed within the frameworks of already existing solutions, offering non-specialist operators of the fake-news detection system insights into the model decision process. Instead of redesigning the model to make it more transparent, explainability can be provided by attaching an additional, convenient, plug-and-play module. The method requires access to the sample, model’s classification function, and the tokenizer.

**Figure 1:**
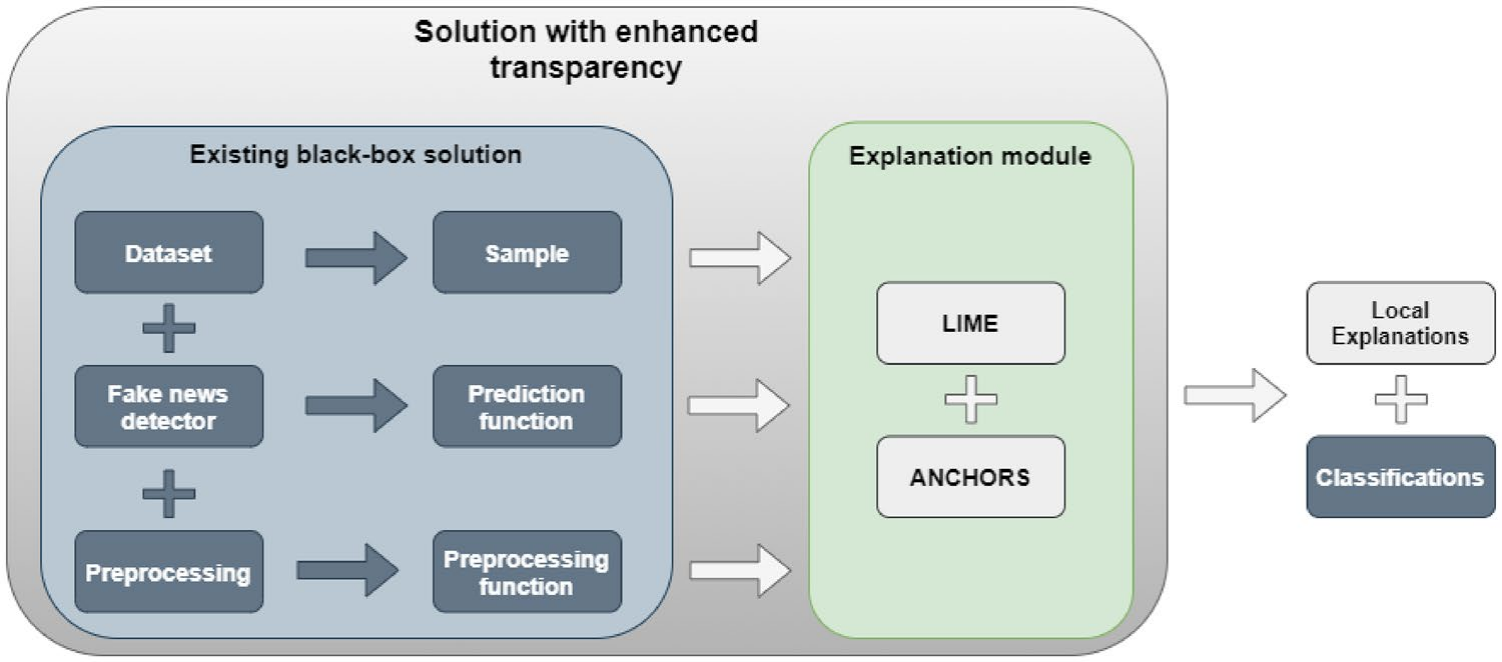
The overview of the proposed approach.

 This central idea is illustrated in Fig. 1. There, the existing black-box solution still operates according to its original design and deployment environment. No retraining or modifications to the model itself are necessary. The Explanation Module can be treated as an extension of the system capabilities and another point in the data processing pipeline, to which the samples are redirected. There, the explanation of a sample can be provided using model-agnostic methods (in this case LIME and Anchors), and thus supplement the final classification with valuable insights.

This advantage comes directly from the usage of surrogate-based explanation method. These algorithms belong to the group of the ’post-hoc’ explainability techniques, i.e., ones that aim to convert an existing opaque model to one with a degree of transparency^36^. Specifically, these kinds of methods represent an approach called ’explanation by the simplification’^36^. They usually use a new, inherently explainable model such as a decision tree to approximate the decisions of the original^46^. These can either provide local or global explanations, where locally explainable methods focus on single data inputs, while global ones explain inputs on the whole domain^34,46^. This work focuses on explaining the decisions on a sentence to sentence basis, and consequently, the local variants were used.

The two chosen surrogate-type explanation methods are LIME and Anchors.

### LIME

LIME^47^ is the model-agnostic method that is easy to interpret and locally faithful, i.e., it represents the model behaviour in the neighbourhood of the predicted sample^47^. LIME, in essence, samples instances around the prediction being explained and perturbs them to train an inherently interpretable linear model. The principle behind this is that any complex model is linear at the local scale and this assumption, in theory, should provide a good local approximation.

The mathematical foundation is explained by the authors in the original paper^47^. The general expression for obtaining an explanation $\xi$ for sample $x$ with LIME is presented in Eq. (1).

1 $$ \xi (x) = \mathop {{\text{arg}}{\mkern 1mu} {\text{min}}}\limits_{{g \in G}} L(f,g,\pi _{x} ) + \Omega (g) $$

Where $G$ is a class of potentially interpretable models, $g \in G$ is a model that can be presented to the user as an explanation and $\Omega (g)$ is a measure of complexity. $L(f, g, \pi _x)$ quantifies how *unfaithful* $g$ is in approximating explained model $f$ in the locality defined by a proximity measure between an instance $z$ and sample $x$ ($\pi _x$)^47^. The goal is to find balance between minimization of $L(f, g, \pi _x)$ and maintaining human understandable levels of $\Omega (g)$^47^.

Since the authors of the method wanted their solution to be model-agnostic^47^, no assumptions about the model $f$ could be done. Instead, the $L(f, g, \pi _x)$ is approximated by drawing samples depending on the proximity $\pi _x$^47^. To quote the authors: “We sample instances around $x'$ by drawing nonzero elements of $x'$ uniformly at random (where the number of such draws is also uniformly sampled). Given a perturbed sample $z' \in \{0, 1\}^{d'}$ (which contains a fraction of the nonzero elements of $x'$), we recover the sample in the original representation $z \in R^d$ and obtain $f(z)$, which is used as a label for the explanation model. Given this dataset $Z$ of the perturbed samples with the associated labels, we optimize Eq. (1) to get an explanation $\xi (x)$.”^47^, p. 3.

The general formulation from Eq. (1) can be used with various explanation algorithms $G$, fidelity functions $L$ and complexity measures $\Omega$. Authors use a combination where $G$ is the class of sparse linear models, $L$ is defined as square loss, and $\pi _x$ is an exponential kernel defined on some distance function $D$^47^. The Algorithm 1 presents algorithmic steps required to obtain an explanation with this approach. 
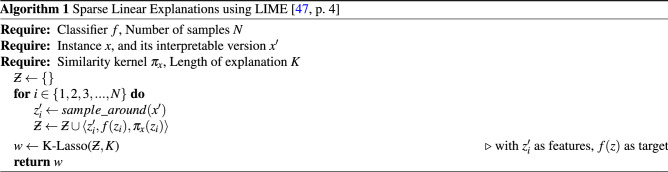


### Anchors

Anchors is a model-agnostic explanation algorithm based on ‘if-then’ rules^48^. Such ‘anchor’ is a rule applied to the local prediction where ‘changes to the rest of the feature values of the instance do not matter’^48^, p. 1. It means that the prediction is always supposed to be the same, for an instance on which the anchor holds^48^. As^48^ highlight, anchors are intuitive, easy to comprehend, and have clear coverage.

The mathematical formulation of anchors was explained in detail by its authors in the original paper^48^. Similarly to the LIME^47^ method, the goal is to explain a *f*(*x*) given the non-transparent model $f: X \rightarrow Y$ and an single instance $x \in X$. To achieve that, the instance *x* must be perturbed using a “*perturbation distribution* *D*”^48^, p. 2 using an interpretable representation. Thus, an *anchor* *A* is an set of feature predicates on *x* that achieves precision greater or equal to some level of precision $\tau$. This can be formally represented with the Eq. (2)^48^, p. 4, where *D*(*z*|*A*) represents the conditional distribution when the rule *A* applies^48^.

2 $$\begin{aligned} prec(A) = \mathbb {E}_{D(z|A)}[\mathbbm {1}_{f(x)=f(z)}] \ge \tau \end{aligned}$$

In case of the text classification, the interpretable representation is made of the individual words from the explained instance *x*, where *D* replaces missing words with random ones of the same Part-of-Speech (POS) tag, based on the probability proportional to their similarity in the embedding space^48,49^.

The search for an anchor is a non-trivial problem, since their number of all possible anchors is exponential and intractable to solve exactly^47^. Furthermore, it not feasible to compute precision from Eq. (2) directly for an arbitrary *D* and *f*^48^. Thus, it has to be redefined in probabilistic terms: “*anchors satisfy the precision constraint with high probability*”^48^, p. 4. This form is shown in the Eq. (3)^48^, p. 4.

3 $$\begin{aligned} P(prec(A) \ge \tau ) \ge 1 - \delta \end{aligned}$$

Thus, the search for an anchor is expressed as combinatorial optimization problem in the equation (4).

4 $$\begin{aligned} \max _{A s.t. (prec(A) \ge \tau ) \ge 1 - \delta }cov(A) \end{aligned}$$

Where *cov*(*A*) represents the coverage of an anchor, defined in the Eq. (5)^48^, p. 4.

5 $$\begin{aligned} cov(A)=\mathbb {E}_{D(z)}[A(z)] \end{aligned}$$

One of the possible algorithmical solutions was proposed by the authors in their original work^48^. Due to the limitations of the greedy approach, such as irreversibility of the suboptimal choices and focus on the length instead of coverage, it was necessary to introduce beam-search. It is performed by guiding the search towards the anchor with the highest coverage over the maintained set of candidate rules. The outline of this approach is shown in algorithm 2, where *B* is a set of current candidates and the *B*-best candidates are kept due to the results from KL-LUCB approach with multiple arms^48,50^. Given a tolerance $$\varepsilon \in [0, 1]$$, it returns a set of candidates size *B* that is highly probable $$\varepsilon $$-approximation of the anchor with the highest true precision $${\mathscr {A}}^*$$^48^. 
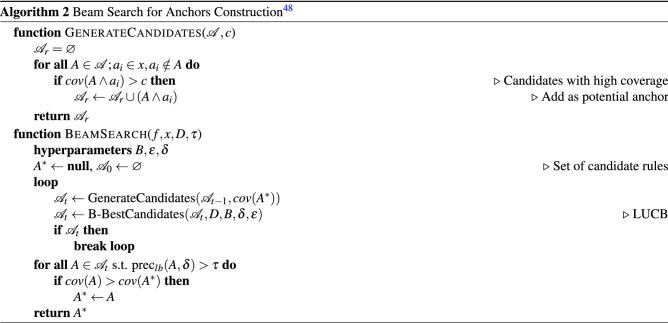


## Methodology

### Experimental process

To verify the usability of the proposed approach on various architectures, experiments were conducted on the two separate models, Bidirectional Long Short Term Memory classifier and BERT-based fake news detector. Moreover, each of them was trained on a separate dataset.

The research procedure consisted of three parts. The first one distilled the datasets into content type expected on the social media platforms. The second one had been the training of the classifiers to distinguish between real and fake news. Finally, there had been two surrogate-type methods chosen to explain the model predictions. Therefore, the general process followed these steps:

1. Initial data preparation,
2. Construction and training of the classifier,
3. Configuration and application of the selected xAI surrogate-type methods.

Data preparation processes are described in subsections ’The data for the Bidirectional LSTM fake news detector’ and ’The data for the BERT-based fake news detector’, the models’ final architectures and training processes in subsections ’Bidirectional Long Short Term Memory classifier architecture and training’ and ’BERT model architecture and training’, while the details of explanation methods are presented in subsections ’LIME’ and ’Anchors’.

Finally, to see what patterns were discovered in the models’ predictions and how they relate to their decisions, the following scenarios were investigated:

1. When the model correctly classified a title as fake news,
2. When the model correctly classified a title as real news,
3. When the model incorrectly classified a title as fake news,
4. When the model incorrectly classified a title as real news.

### Bidirectional long short term memory classifier architecture and training

For the Bidirectional Long Short Term Memory (LSTM) classifier to work efficiently, text was first converted to the lower case, cleaned of stopwords, stemmed, one-hot encoded and embedded using methods from keras and nltk modules.

The used model is comprised of the Embedding Layer, Bidirectional LSTM layer, and Dense output layer. The input_dim of Embedding Layer is set to 20 000, output_dim to 40, and input_length to 20. Bidirectional LSTM layer has 100 neurons, while the Dense output layer has two outputs and uses Softmax. Dropout is set through the network to 0.7.

The model uses Adam as the optimiser, while Sparse Categorical Crossentropy serves as the loss function. The utilised metric is Sparse Categorical Accuracy. Batch size is 64, while tests have proven that five epochs are enough for the data used. Additionally, early stopping is employed with patience set to two, monitoring Sparse Categorical Accuracy. All other parameters that were not mentioned are set to default.

###  BERT model architecture and training

Before the actual training, BERT models need each input sentence to be transformed by a tokenizer. Firstly, the tokenizer breaks words into tokens. Then it adds unique [CLS] and [SEP] tokens at the beginning and the end of the sentence accordingly. Lastly, the tokenizer replaces each token with the corresponding id. The id comes from the pre-trained embedding table. The reasons behind this process and further details are presented by Horev^51^. The tokenizer was configured to either truncate or pad data to the ‘max length’. In this case, this parameter is set to 59, appropriately to the demands of short titles and Twitter’s character cap. Additionally, everything is converted to the lower case.

DistilBERT^52^ is the BERT variant employed in this study. It is a lighter and faster version of the original BERT, which retains similar performance, developed by the team at ’HuggingFace’. The used model imposes tokenizer selection since those two must match to work correctly.

Transfer learning^53^ is employed to create an effective model quickly. It means already pre-trained distilBERT is used within the model as a layer frozen during the training process. The only layers that are being optimised are those added to the distilBERT to perform classification. One LSTM layer, one pooling layer, one dense feedforward layer and an output layer.

The LSTM layer comprises 50 units with the activation function being Hyperbolic Tangent and recurrent activation function being Sigmoid. Furthermore, this layer had both dropout and recurrent dropout set to 0.1. Pooling layer is a default instance from tf.keras.layers.GlobalMaxPool1D. The dense feedforward layer also had 50 units, used Rectified Linear Unit (ReLU) as the activation function, and dropout equal to 0.2. The final layer had only two units and employed Softmax.

This model also uses Adam as the optimiser and Sparse Categorical Crossentropy as the loss function. The utilised metric is again Sparse Categorical Accuracy. Batch size is 100, while tests have proven that three epochs are enough in this case. Furthermore, early stopping is employed here as well, with patience set to two, monitoring Sparse Categorical Accuracy.

### Configuration of the selected xAI methods

A version of LIME designed to work with text was employed. It was configured to present the top five features and to use 5000 samples in the neighbourhood to train a local linear model.

Anchors had to use Spacy Object, described more in the Technology Stack subsection, to perform textual explanations. Default trained pipeline package, ‘en_core_web_sm’ had been downloaded and used. Attribute ‘threshold’ was set to 95%, ‘temperature’ to 0.3, ‘beam_size’ to three, and ‘top_n’ to 1000. Examples shown were set to be perturbed by replacing words with ’UNKs’.

Both algorithms needed auxiliary functions, which tokenize the text and return model prediction. Explanations were derived on the test set expanded with the model’s predictions.

### Technology stack

NumPy^54^ and Pandas are both standard modules for any data science and machine learning tasks. NumPy offers a multidimensional array object and its derivatives supported with a selection of optimised routines for fast operations. On the other hand, Pandas provides unique structures and operations for convenient data handling, making work with tables or time series much more manageable.

Scikit-learn^55^ is an open-source collection of various machine learning algorithms and auxiliary methods, such as metrics, data normalisation, dataset splitting, and more.

For the construction of the neural network, Tensorflow^56^ together with Keras were used. Tensorflow is an open-source platform for machine learning, where Keras is an API standard for defining and training neural networks. The Tensorflow-GPU is used to perform computation in the computer’s graphical processing unit. For this purpose, CUDA^57^ had to be also utilised.

The tokenizer and the pre-trained distilBERT model used in this study come from the Transformers module from HuggingFace Team^58^. It is an open-source library with the selection of modern transformers architectures under a unified API^58^.

Anchor version used in the study comes from Alibi library.

As mentioned in the subsection dedicated explainability techniques, for Anchors to work with textual data, the SpaCy object is necessary. SpaCy is an open-source library designed for advanced Natural Language Processing in Python. It offers most language processing-related features. In this study, its use is limited to the delivery of the trained pipeline package. It usually contains tokenizer, tagger, parser, entity recognizer, and lemmatizer.

LIME explanation algorithm was included in a separate package made available by the author on PyPi package manager.

## Results

### The data for the Bidirectional LSTM fake news detector

The dataset used for this model comes from the competition hosted on the data science portal ’kaggle.com’. For the experiment file “train.csv” was used, since it comes with labels. It has over 20.8k samples, but after the removal of rows with NaN values 18 285 samples remains, in which 10 361 data points represent real news and 7 924 represent fake news. It originally comes with columns id, title, author, text, and label. However, only the ’label’ and ’title’ were used to represent content on the social media platforms.

The dataset was split into the training and test portion with stratification, where 77% of all samples ended up in the training subset.

Moreover, after the model had finished the classification task, the test data was expanded with an additional column containing the model predictions. It was done to be later able to choose suitable samples for each test scenario.

### The data for the BERT-based fake news detector

Currently, the authors of this work are implementing a solution that will accept input data from the user. The results of this work are part of the H2020 SocialTruth project. For the sake of replicability, this research is presented using a well-known benchmark dataset.

The dataset used for this model is publicly available on the portal ’kaggle.com’^59^ and originally comes from the work of Ahmed, H., Traore, I. & Saad, S.^60^. The authors took genuine news articles from the news website ’Reuters.com’, while the fake ones were collected from another dataset on the portal ’kaggle.com’.

The dataset is split initially into two Coma-Separated Values (CSV) files. One is for the verified news, with 21 417 samples, and the other for the fake ones, with 23 481 samples. Those separate files had to be merged and reshuffled.

Four attributes describe each sample: the title, the text of the article, the subject of the article, and the date of publication. Since the purpose of this work was to simulate the content present on social media platforms such as Twitter, of the four attributes, only the ‘title’ had been used. The dependent variable had to be manually added to the dataset. The dataset was split into the training and test portion, with 80% of all samples belonging to the training.

As it was the case with data for the Bidirectional LSTM classifier, after the model had finished the classification task, the test data was expanded with an additional column containing the model predictions.

### Evaluation metrics

For the purpose of the model’s evaluation, following metrics were utilised:

1. **Precision**—The ratio of actual fake news detected by the model and all the news classified by the model as fake. In terms of the true positives (TP) and false positives (FP), precision (*p*) can be formulated as the equation (6)^61^.
  6 $$\begin{aligned} p=\frac{TP}{TP+FP} \end{aligned}$$
2. **Recall**—The ratio of actual fake news detected by the model and all the fake news present in the dataset. In terms of the true positives (TP) and false negatives (FN), recall (*r*) can be formulated as the equation (7)^61^.
  7 $$\begin{aligned} r=\frac{TP}{TP+FN} \end{aligned}$$
3. **F1-Score**—A harmonic average of the precision (*p*) and recall scores (*r*), defined as the equation (8)^61^.
  8 $$\begin{aligned} F_1=2\cdot \frac{r\cdot p}{r+p} \end{aligned}$$

### Classification results

Table 1 shows the results for BERT-based fake news detector and Bidirectional LSTM fake news detector (BI-LSTM) achieved on their respective test datasets.

BERT-based fake news detector has accuracy of 98%. Precision for real news is 97%, while for fake news it is equal to 99%. The recall is reversed, with the 99% score for the real news and the 97% for fake news, while f1-score for both classes is 98%. Additionally, the last column, *“support”*, presents the number of samples representing each category within the test subset.

Bidirectional LSTM has an accuracy of 92%. The precision for real news is 94%, while for fake news it is equal to 90%. The recall equal to 92% is the same for both categories. The f1-score achieved for the real news classes is 93% and 91% for fake news. The last column, *“support”* once again, presents the number of samples representing each category within the test subset.

In summary, both models achieved promising results on their respective datasets based on their architecture, making them viable for the next stages of the experiment.

**Table 1:** Models’ classification results of fake news.

Model	Data	Precision (%)	Recall (%)	F1-score (%)	Support
BERT	Real	99	98	98	4328
	Fake	98	99	98	4652
BI-LSTM	Real	94	92	93	3420
	Fake	90	92	91	2615

### Output of surrogate-type explanations for BERT-based fake news detector

Explanations acquired from the chosen explanation methods for the first four scenarios are listed in Tables 2 and 3 . The first contains the LIME results, while the second presents the Anchors output. The table with LIME results has a row for each of the first four test scenarios. Every row also has the used sentence, the model’s prediction probabilities, words highlighted by the method, and their weights, representing impact on the prediction probability. The ‘+’ sign means that the weight increases the chance of the sentence being fake, while the sign ‘-’ marks the opposite.

The table with the Anchors output also has a row for each of the first four scenarios and the text of the used sentence. Precision is best explained by the example. For instance, in the third row, precision is equal to 0.96. It is the probability at least 0.96 high that each perturbed instance of the sentence ’Trump looms behind both Obama and Haley speeches’ where words ‘and’ and ‘Obama’ are present, will be classified as fake news. Anchors are the words compromising the ‘if-then’ rule around which the explanation is built.

The first test case was when the model correctly classified a title as fake news. Since the model had fared well in separating fake from real news, such examples were abundant. The picked instance for this case was ‘FBI NEW YORK FIELD OFFICE Just Gave A Wake Up Call To Hillary Clinton’.

In this instance, the model is sure about the falsehood of the title, and it is worth pointing out words that were spotlighted there. These are ‘Hillary Clinton’ and the phrase ‘Just gave a’.

The Anchors in this study were sometimes unable to find ‘if-then’ rules. It occurred in this scenario and is very common to all titles classified as fake.

The second test case was when the model correctly classified a title as real news.

The selected title was ‘Turkey-backed rebels in Syria put IS jihadists through rehab’. LIME explanation shows the model’s high confidence in this instance as well. However, the found words seem to have meagre weights compared to those in the previous test. Moreover, it seems that the model concentrates on designations, such as ‘Turkey’, ‘Syria’, and ‘jihadists’.

In this test, Anchors did successfully find the ‘if-then’ rule. Here, ‘anchor’ is the combination of words ’rehab’, ‘Turkey’, and ‘through’. Moreover, looking at the precision equal to 1.00, when these three appear together, the model’s prediction is always ‘real’. The words ’rehab’ and ‘through’ may be just the result of an algorithm search without much meaning behind it. What is crucial is that ‘Turkey’ overlaps with LIME explanations, which is a strong indicator of its importance.

The sentence ‘Trump looms behind both Obama and Haley speeches’ was used in the third case. In this instance, the LIME explanation shows that the model has assigned relatively similar probabilities to both target classes, with a relatively moderate advantage of 0.16 to the ‘fake’ category. It seems this comes from the presence of the names ‘Obama’ and ‘Haley’. The model’s prediction might have been influenced by the fact that part of fake news in the dataset had concerned those persons.

This notion is further reinforced by the output of anchors for this sentence. The name ‘Obama’ is part of the ‘anchor’, overlapping with LIME explanations. This occurrence develops the idea that names of characters or places that are the subject of fake news often can have an impact on the model’s decisions.

The fourth test scenario is depicted with the sentence’ Pope Francis Demands Christians Apologize For Marginalizing LGBT People’. LIME’s explanation shows that the model was reasonably sure to consider this title ‘real’. Based on it, no strong patterns suggested its ‘fakeness’ with marginal weights of words that could change it. Perhaps it is a matter of the dataset, where similar combinations appear rarely.

Looking at the parallel row in Table 3, it is notable that an extensive ‘anchor’ was necessary. It encompasses almost every word except for ‘Marginalizing LGBT People’. Those seem to have no impact, and according to this explanation, could not influence the outcome.

**Table 2:** LIME explanations for the four test scenarios - BERT-based classifier.

Test	Sentence	Probability fake	Probability real	Highlighted words	Weights
1	FBI NEW YORK FIELD OFFICE Just Gave A Wake Up Call To Hillary Clinton	0.97	0.03	1. Gave 2. Just 3. A 4. Hillary 5. Clinton	+ 0.28 + 0.25 + 0.23 + 0.21 + 0.17
2	Turkey-backed rebels in Syria put IS jihadists through rehab	0.00	1.00	1. Turkey 2. Syria 3. in 4. backed 5. jihadist	– 0.02 – 0.02 – 0.01 – 0.01 – 0.01
3	Trump looms behind both Obama and Haley speeches	0.58	0.42	1. and 2. Obama 3. Haley 4. looms 5. behind	+ 0.17 + 0.16 + 0.13 – 0.05 + 0.05
4	Pope Francis Demands Christians Apologize For Marginalizing LGBT People	0.29	0.71	1. For 2. Pope 3. People 4. Marginalizing 5. LGBT	– 0.10 – 0.08 + 0.08 + 0.08 + 0.04

**Table 3:** Anchors explanations for the four test scenarios—BERT-based classifier.

Test scenario	Sentence	Precision	Anchors
Correctly classified fake news	FBI NEW YORK FIELD OFFICE Just Gave A WakeUp Call To Hillary Clinton	–	Anchors not found
Correctly classified real news	Turkey-backed rebels in Syria put IS jihadists through rehab	1.00	rehab AND Turkey AND through
Misclassified real news as fake news	Trump looms behind both Obama and Haley speeches	0.96	and AND Obama
Misclassified fake news as real news	Pope Francis Demands Christians Apologize For Marginalizing LGBT People	1.00	Francis AND POPE AND whitespace AND For AND Apologize AND Christians AND Demands

### Output of surrogate-type explanations for Bidirectional LSTM fake news detector

Analogously to the previous subsection, explanations acquired with the chosen explanation methods for the first four scenarios are listed in Tables 4 and 5 , where the first contains the results of LIME, while the second presents the Anchors output.

The first correctly classified fake news sample was ’Wikileaks List Exposes at Least 65 Corporate ‘Presstitutes’ Who Colluded to Hide Clinton’s Crimes’. LIME’s output demonstrates that there was no ambiguity and the sample was classified as fake with full confidence. The most impactful words for the model’s prediction were ’Exposes’, ’Wikileaks’ and ’Presstitutes’. The last word, ’Presstitutes’, was also present in the output of Anchors, suggesting its importance. This is in line with expectations, since such emotionally charged terms are more common for the fake content and ’click-bait’ titles. Therefore, by comparing the outputs from both methods, a user can gain an insight into the reasons behind the model decision.

According to the LIME’s explanation, the second test sample has 100% probability of being real. The title “Senate Formally Takes Up Gorsuch Nomination, and Braces for Turmoil - The New York Times” was correctly recognised as representing the real news. It is worth noting, that from the five highlighted words, three are names. Their impact is significant, with their summed weights equal to 0.32. There was no explanation from Anchors for this case.

In the third test case model was incorrect, and the real news was classified as fake. Looking at the probabilities provided by LIME, the model’s uncertainty was minimal. Assigned probability that the prediction is fake is equal to 95%. While the weights of the particular words are relatively low, comparison with Anchors’ output suggests high impact of the names on the models classification results.

Lastly, the model classified the fake news title ’New Alleged Audio: Bill Clinton Encourages Mistress to Hide His Role in Securing Her a State Job’ as real news. Analysis of the provided explanations delivers an indication of the words ’Securing’, ’Role’ and ’Bill’ as crucial, which outweighed two words that could be associated with fake news, that is ’Mistress’ and ’Clinton’.

**Table 4:** LIME explanations for the four test scenarios - Bidirectional LSTM classifier.

Test	Sentence	Probability fake	Probability real	Highlighted words	Weights
1	Wikileaks List Exposes at Least 65 Corporate ‘Presstitutes’ Who Colluded to Hide Clinton’s Crimes	1.00	0.00	1. Exposes 2. Wikileaks 3. Presstitutes 4. Colluded 5. Least	+ 0.02 + 0.02 + 0.01 – 0.01 – 0.01
2	Senate Formally Takes Up Gorsuch Nomination, and Braces for Turmoil - The New York Times	0.00	1.00	1. York 2. Gorsuch 3. Nomination 4. Times 5. Turmoil	– 0.13 – 0.12 – 0.09 – 0.07 – 0.05
3	Alyssa Milano: Up to Women to Remove Trump from Office	0.95	0.05	1. Milano 2. Trump 3. Alyssa 4. Remove 5. Office	+ 0.07 – 0.04 – 0.03 – 0.03 – 0.03
4	New Alleged Audio: Bill Clinton Encourages Mistress to Hide His Role in Securing Her a State Job	0.11	0.89	1. Securing 2. Role 3. Bill 4. Mistress 5. Clinton	– 0.48 – 0.42 – 0.20 + 0.13 + 0.11

**Table 5:** Anchors explanations for the four test scenarios - Bidirectional LSTM classifier.

Test scenario	Sentence	Precision	Anchors
Correctly classified fake news	Wikileaks List Exposes at Least 65 Corporate ‘Presstitutes’ Who Colluded to Hide Clinton’s Crimes	0.96	Clinton AND Presstitutes
Correctly classified real news	Senate Formally Takes Up Gorsuch Nomination, and Braces for Turmoil - The New York Times	–	Anchors not found
Misclassified real news as fake news	Alyssa Milano: Up to Women to Remove Trump from Office	0.98	Milano AND Women AND UP
Misclassified fake news as real news	New Alleged Audio: Bill Clinton Encourages Mistress to Hide His Role in Securing Her a State Job	1.00	Role

## Conclusion

The achieved results allow to draw three significant conclusions. To begin with, it is possible to use surrogate-type methods to explain locally opaque fake news detectors, including BERT-based models, to a degree. As there is a body of research dedicated to using BERT-based methods for fake news detection, using surrogate explainability methods, as proposed in this paper, can be of significant value to the operators of the system. The methods can capture meaningful patterns driving model behaviour. The application is straightforward and convenient with a ’plug-and-play’ approach. Furthermore, they are easy to use and understand, offering value for both the user and developer and can be applied to a plethora of models. Nevertheless, the results confirm that more than one surrogate-type method should be used to derive explanations, as it can be seen that various techniques tend to highlight distinct patterns. Some do overlap. Additionally, there remains an issue of Anchors not always being able to find an explanation.

The experiments show that the explanation mechanism can benefit from employing diverse methods to better highlight meaningful patterns.

In this work, the application of surrogate-type explainability techniques to the linguistic-based approach to fake news detection was investigated. Particularly, when using both a fine-tuned BERT and a Bidirectional LSTM to assess the veracity of short pieces of text, this study has verified the validity of the surrogate-type explanation methods usage.

## Data Availability

The datasets used in this research are open benchmark datasets provided by the portal ’kaggle.com’^59^ and are available for download at https://www.kaggle.com/clmentbisaillon/fake-and-real-news-dataset/activity and https://www.kaggle.com/c/fake-news/data?select=train.csv.
